# Comparison of posterior correction results between Marfan syndrome scoliosis and adolescent idiopathic scoliosis—a retrospective case-series study

**DOI:** 10.1186/s13018-015-0210-z

**Published:** 2015-05-21

**Authors:** Weiqiang Liang, Bin Yu, Yipeng Wang, Zhengyao Li, Guixing Qiu, Jianxiong Shen, Jianguo Zhang

**Affiliations:** Department of Orthopedics, Peking Union Medical College Hospital, Chinese Academy of Medical Sciences and Peking Union Medical College, 1 shuai fu yuan, wang fu jing street, Dong Cheng District, Beijing, 100730 People’s Republic of China

**Keywords:** Idiopathic scoliosis, Marfan syndrome, Surgery

## Abstract

**Background:**

The X-ray films of the patients with Marfan syndrome scoliosis (MSS) look like those with adolescent idiopathic scoliosis (AIS). In literature, there are many reports on the correction results of AIS, while there are a few studies focused on the difference of the correction results between MSS and AIS. This study aims to analyze whether there are differences of posterior correction surgery in MSS and AIS.

**Methods:**

All the patients included underwent posterior correction surgery. The radiographic data, operation duration, estimated blood loss, transfusion, fusion levels, and correction rate were retrospectively reviewed and analyzed between the two groups.

**Results:**

Group MSS included 42 patients, 11 male and 31 female, with an average age of 15.2 years old. Group AIS included 168 patients (ratio, 1:4), 34 male and 134 female, with an average age of 14.5 years old. Twenty-three patients in group MSS and 94 patients in group AIS were followed up regularly, with an average time of 18.4 and 18.5 months, respectively. The mean coronal Cobb angle of the major curve before operation and at final follow-up, the correction rate, fusion level, operation duration, estimated blood loss during operation, and transfusion between the two groups were 60.4 and 57.5°, 14.6 and 15.2°, 76.4 and 74.1 %, 11.5 and 11.0 vertebrae, 4.6 and 4.0 h, 845 and 698 ml, and 1151 and 894 ml, respectively. The age, gender ratio, curve type, and coronal Cobb angle of the major curve were all matched (all *P* > 0.05). Group MSS had a longer operation duration and more estimated blood loss compared with those of group AIS (both *P* < 0.05), while there was no significant difference in terms of fusion level, transfusion, coronal Cobb angle of the major curve at final follow-up, and the correction rate (all *P* > 0.05).

**Conclusions:**

When performing posterior correction for scoliosis, the surgeons should be aware that the patients with Marfan syndrome scoliosis had more estimated blood loss and longer operation duration than AIS patients, while the correction rate was similar.

## Background

Marfan syndrome (MFS) is an autosomal dominant disorder of connective tissue, and the ocular, cardiovascular, and skeletal system involvements are the cardinal features. In literature, 60 % of patients with Marfan syndrome have scoliosis, and approximately one quarter to one half of those patients have curves severe enough to consider surgical correction, despite non-operative measures [1, 2]. On anteroposterior and lateral X-ray films of the full spine, the patients with Marfan syndrome scoliosis (MSS) do not have failure of formation or failure of segmentation of the vertebra, thus look like idiopathic scoliosis. To our knowledge, there are a few studies focused on the comparison of the correction results between MSS and adolescent idiopathic scoliosis (AIS) [3], so we reviewed our patients with MSS or AIS and compared whether there were differences in the correction.

## Materials and methods

### General data

Institutional review board (IRB) approval of Peking Union Medical College hospital was obtained before the study. Then, we retrospectively reviewed the medical records and radiographic data of the patients that underwent correction surgery with MSS or AIS in our hospital from January 2002 to December 2012. The diagnosis of Marfan syndrome was according to the Ghent criteria [4]. The operation duration, estimated blood loss (EBL) and transfusion, radiographic parameters of the spinal deformity, fusion level, correction rate, and body height change were recorded. The inclusion criteria were as follows: (1) age between 10 and 20 years old; (2) the Cobb angle of the major curve was 40~100°; (3) single posterior correction and fusion surgery, no osteotomy or anterior surgery; (4) the instruments were all third-generation correction system with hook-hook, screw-hook, or screw only fixation fashion.

Forty-two MSS patients were included, 11 male and 31 female, with an average age of 15.2 years old (range, 10 to 20). Among the patients, 10 had a single thoracic or thoracolumbar curve, 7 had double thoracic curves, 17 had thoracic and lumbar curves, and 8 had triple curves. The average Cobb angle of the major curve was 60.4° (range, 41~98°) and the flexibility of the major curve was 53.6 % (range, 14.3~100 %). Twenty-three patients were followed up regularly, 7 male and 16 female, with an average follow-up time of 18.4 months (range, 6 to 69 months). Among the 23 patients, 6 had a single thoracic or thoracolumbar curve, 1 had double thoracic curves, 10 had thoracic and lumbar curves, and 6 had triple curves.

AIS patients were matched at a 1:4 ratio. The patients were matched according to the curve type; the first 50 % cases and the last 50 % cases in the database were selected. One hundred and sixty-eight AIS patients were included, 34 male and 134 female, with an average age of 14.5 years old (range, 10 to 20). Among the patients, 40 had a single thoracic or thoracolumbar curve, 28 had double thoracic curves, 68 had thoracic and lumbar curves, and 32 had triple curves. The average Cobb angle of the major curve was 57.5° (range, 42~96°) and the flexibility of the major curve was 53.3 % (range, 6.7~100 %). Ninety-four patients were followed up regularly, 21 male and 73 female, with an average follow-up time of 18.5 months (range, 6 to 72 months). Among the 94 patients, 20 had a single thoracic or thoracolumbar curve, 15 had double thoracic curves, 39 had thoracic and lumbar curves, and 20 had triple curves.

The age, gender ratio, curve type, and the preoperative Cobb angle of the major curve were all matched (all *P* > 0.05).

### Measurements of the X-ray films

The standing anteroposterior (AP) and lateral X-ray films of the full spine before operation, after operation, and at final follow-up, and preoperative supine Bending films were reviewed and measured. The coronal Cobb angle and apical vertebral translation (AVT) of the major curve, coronal trunk balance (CTB), and sagittal thoracic kyphosis and lumbar lordosis before operation, after operation, and at final follow-up were also recorded. The AVT was defined as the vertical distance between the center of the apex to the plumb line of C7 on the standing AP film (thoracic curve) or the center sacral vertical line (thoracolumbar/lumbar curve) (mm) [5]. Sagittal thoracic kyphosis was measured from T5 to T12, and lumbar lordosis was measured from L1 to S1 on lateral view. The CTB was defined as the vertical distance from the center of S1 to the plumb line of C7 on the standing AP film (mm) [5]. Post-operative decompensation was defined as coronal trunk balance over 20 mm, lumbar curve deterioration in the coronal plane, or junctional kyphosis (T12-L1) larger than 10° on sagittal plane [6].

### Statistics

SPSS 17.0 for Windows (SPSS, Inc., Chicago, IL) was used for statistical analysis. The clinical, operative, and radiographic variables of each group were compared using student *t* test or Mann–Whitney *U* test and chi-square test. A *P* value lower than 0.05 was considered as significant.

## Results

The fusion level, operation duration, EBL, and transfusion are listed in Table 1. The patients in the MSS group had a longer operation duration, more EBL per vertebra fused, and more auto-transfusion than those in group AIS (all *P* < 0.05), while there was no significant difference in terms of fusion level, total transfusion, or body height change between the two groups (all *P* > 0.05). Compared with those in group AIS, the patients in group MSS also had a higher incidence of whole blood transfusion (*P* < 0.05), while the incidences of RBC transfusion and plasma transfusion were similar (*P* > 0.05).

**Table 1:** Demographic data between group MSS and group AIS (x ± SD)

	Group MSS (*n* = 42)	Group AIS (*n* = 168)
Age (year)	15.2 (10~20)	14.5 (10~20)
Gender ratio (M:F)	11:31	34:134
Fusion level (vertebra)	11.5 ± 2.8 (5~15)	11.0 ± 2.7 (5~16)
Operation duration (hour)	4.6 ± 1.5 (2.2~9)	4.0 ± 1.1 (2.2~8)*
Total EBL (ml)	845 ± 441 (300~2500)	698 ± 458 (200~5000)*
EBL per vertebra fused (ml)	74.6 ± 37.2 (29~208)	63.1 ± 31.7 (20~313)*
Auto-transfusion (ml)	487 ± 318 (33 cases) (100~1300)	367 ± 233 (136 cases) (100~1900)*
RBC transfusion (U)	3.5 ± 2.1 (13 cases) (2~9)	2.8 ± 1.2 (63 cases) (2~8)
Whole blood transfusion (ml)	700 ± 316 (10 cases) (400~1200)	511 ± 219 (18 cases) (200~1000)
Plasma transfusion (ml)	475 ± 237 (8 cases) (200~800)	385 ± 112 (26 cases) (200~800)
Total transfusion (ml)	1151 ± 1018 (39 cases) (100~4640)	894 ± 790 (156 cases) (100~4847)
Body height change (cm)	4.5 ± 2.4 (1~12)	4.1 ± 1.6 (1~10)

*Compared between group MSS and group AIS, *P* < 0.05

The coronal Cobb angle and AVT of the major curve, CTB, and correction rate of group MSS and group AIS are listed in Table 2. After operation, the coronal Cobb angle and AVT were significantly corrected in both groups (both *P* < 0.05). In group MSS, there was no significant difference of the CTB after operation compared with that of pre-operation (*P* = 0.207). Compared with that of post-operation, there was no significant difference of the coronal Cobb angle, correction rate, AVT, or CTB at final follow-up in this group (all *P* > 0.05). As for group AIS, CTB was significantly corrected (*P* < 0.05) and was further corrected at final follow-up (*P* < 0.05). At final follow-up, the coronal Cobb angle became larger and the correction rate turned smaller (both *P* < 0.05), while not for AVT (*P* > 0.05) compared with those of post-operation.

**Table 2:** Correction of the coronal parameters of the major curve between group MSS and group AIS (x ± SD)

	Pre-OP		Post-OP		Final F/U	
	Group MSS (*n* = 42)	Group AIS (*n* = 168)	Group MSS (*n* = 42)	Group AIS (*n* = 168)	Group MSS (*n* = 23)	Group AIS (*n* = 94)
Coronal Cobb angle (degree)	60.4 ± 16.3 (41~98)	57.5 ± 12.6 (42~96)	13.7 ± 9.7 (1~42)	12.7 ± 8.7 (0~47)	14.6 ± 9.0 (4~36)	15.2 ± 8.7 (0~47)
Correction rate (%)			78.7 ± 11.9 (46.1~98)	78.7 ± 12.3 (44.4~100)	76.4 ± 11.1 (53.5~90.9)	74.1 ± 12.4 (43.2~100)
AVT (cm)	4.9 ± 2.4 (0.4~12)	4.1 ± 1.8 (0.6~9.3)	1.5 ± 1.2 (0~5)	1.1 ± 1.0 (0~5)	1.5 ± 0.9 (0.3~4.5)	0.9 ± 0.8* (0~5)
CTB (cm)	1.4 ± 1.0 (0~3.5)	1.3 ± 1.2 (0~6.5)	1.2 ± 1.1 (0~5)	0.8 ± 0.9* (0~6.2)	0.7 ± 0.9 (0~4)	0.4 ± 0.4 (0~2.2)

*Pre-OP* preoperative, *Post-OP* post-operative, *F/U* follow-up, *MSS* Marfan syndrome scoliosis, *AIS* adolescent idiopathic scoliosis, *AVT* apical vertebral translation, *CTB* coronal trunk balance

*Compared between group MSS and group AIS, *P* < 0.05

Between the two groups, group AIS had a lesser CTB (*P* < 0.05), while there was no significant difference of the coronal Cobb angle, correction rate, and AVT (all *P* > 0.05) in terms of the parameters after operation. At final follow-up, group AIS had a lesser AVT (*P* < 0.05), while no significant difference was found in terms of the coronal Cobb angle, correction rate, and CTB (all *P* > 0.05).

Eight cases in group MSS and 15 cases in group AIS suffered from post-operative coronal trunk decompensation (19.0 %, 8/42 vs. 8.9 %, 15/168). At final follow-up, the number changed to 2 cases in group MSS and 1 case in group AIS (8.7 %, 2/23 vs. 1.1 %, 1/94). There was no significant difference of the incidence of coronal trunk decompensation either after operation or at final follow-up (both *P* > 0.05).

The thoracic kyphosis and lumbar lordosis before operation, after operation, and at final follow-up are listed in Table 3. There was no significant difference of the changes of thoracic kyphosis or lumbar lordosis in both groups (all *P* > 0.05). There was no significant difference of the parameters before operation, after operation, and at final follow-up between the two groups, too (all *P* > 0.05). No patient had sagittal decompensation in either group.

**Table 3:** Sagittal parameter changes of the main thoracic kyphosis and lumbar lordosis between group MSS and group AIS (x ± SD)

	Group MSS	Group AIS
Pre-OP thoracic kyphosis (degree)	20.2 ± 18.7 (−20~77)	19.5 ± 15.7 (−23~60)
Post-OP thoracic kyphosis (degree)	19.8 ± 9.9 (1~50)	20.7 ± 8.3 (0~45)
Thoracic kyphosis at final F/U	20.5 ± 8.9 (5~35)	21.1 ± 8.0 (5~50)
Pre-OP lumbar lordosis (degree)	41.5 ± 14.8 (−12~74)	43.3 ± 11.8 (4~72)
Post-OP lumbar lordosis (degree)	41.1 ± 8.0 (25~56)	42.7 ± 8.7 (12~66)
Lumbar lordosis at final F/U	42.6 ± 7.6 (25~55)	41.4 ± 7.0 (16~60)

*Pre-OP* preoperative, *Post-OP* post-operative, *F/U* follow-up

Complications occurred in both groups are listed in Table 4, and there was no significant difference of the complication rate between the two groups (4.8 %, 2/42 vs. 2.4 %, 4/168, *P* = 0.345).

**Table 4:** Complications in both groups

	Gender	Age	Group	Details of complications	Treatment	Prognosis
1	F	17	MSS	Abnormal position of pedicle screws with incomplete paraplegia	Revision surgery	Completely recovered
2	F	13	MSS	Pleural effusion	Observation	Good
3	F	16	AIS	Pull-out of pedicle screw	Revision surgery	Good
4	F	12	AIS	Lumbar curve decompensation after selective thoracic fusion	Revision surgery with fusion of the lumbar curve	Good
5	F	14	AIS	Pulmonary infection	Antibiotics	Good
6	F	11	AIS	Poor wound healing	Wound care	Good

## Discussion

Marfan syndrome is an autosomal dominant connective-tissue disorder affecting the cardiovascular, ocular, and skeletal system. Spinal deformities, such as scoliosis, kyphosis, and flat back are the common features when the skeletal system is involved.

Some authors had reported that the patients with Marfan syndrome had more blood loss during spinal deformity correction surgery [7–9]. In 2002, Lipton et al. reported 23 cases with MSS, and the mean blood loss during correction was 1300 ml (range, 325 ~ 2150 ml) [7]. In Jones et al.’s series, the mean blood loss was 2148 ml (range, 300 ~ 6500 ml) for all 26 MSS patients and 2150 ml for the patients with posterior approach. While in idiopathic scoliosis patients, it would be only 800 to 1400 ml [8]. Di Silvestre also reported 23 MSS cases, and the mean blood loss during correction surgery was 1850 ml (range, 950 ~ 2850 ml) [9]. Zenner et al. also reported 23 cases of MMS, with an average blood loss of 1748 ml (range, 500–3000 ml) in posterior approach [10]. However, some authors reported some studies in different conditions [3, 11, 12]. In 2011, Dai et al. reported 12 MSS patients with a mean blood loss of 690 ml (range, 550 ~ 920 ml) using posterior only approach, and the auto-transfusion with cell saver was 370 ml (range, 180 ~ 410 ml) [11]. In 2012, Gjolaj et al. compared 34 MSS patients with 68 AIS patients and the blood loss during correction surgery were 1700 ml and 1200 ml in total and 164 ml and 136 ml per vertebra fused, respectively, and there were no significant differences (both *P* > 0.05) [3]. In the current study, the MSS patients had more blood loss than the AIS patients (845 ml vs. 698 ml, *P* < 0.05) and also the blood loss per vertebra fused (*P* < 0.05). The MSS patients in the current study had more auto-transfusion than those of AIS and more auto-transfusion than those of Dai’s report (487 ml vs. 370 ml), too. The blood loss in total and per vertebra fused in the current study was less than those of Gjolaj et al.’s report [3].

Di Silvestre et al. reported an average operation duration of 330 min (range, 180 ~ 450 min) [9]. In our patients, the MSS patients had longer operation duration (4.6 h) compared with that of AIS, which was shorter than that of Di Silvestre et al.’s report, while similar to that of Dai et al.’s and Zenner et al.’s [9–11]. Since we did not record the exact time duration according to different steps of the surgery, we are not quite sure which step was the most time-consuming part. However, we believe that the prolonged surgical time might be multifactorial, and the excessive intraoperative bleeding might be one key factor.

Some authors had reported that when performing correction surgery, the MSS patients usually had a longer fusion range [3, 7, 8]. In Lipton et al.’s 23 MSS cases, the average fusion range was 11 vertebrae (range, 7 ~ 16). In their study, none of the 7 cases with both curves fused suffered from larger than 10° progression, while in their 16 cases with secondary curve partially fused, 11 cases suffered from larger than 10° progression. So, Lipton et al. suggested fusing both the primary and secondary curves in MSS patients [7]. Jones et al. analyzed their 26 MSS patients and suggested that the curve with larger than 30° should be fused, and selective fusion in double curve usually was not sufficient [8]. In Gjolaj et al.’s report, there was 1 patient with fusion to S1, 1 patient to S2, and 7 to pelvis, whereas in the matched control AIS group, no patients required fusions to the pelvis primarily or secondarily. They found that the MSS group had a longer fusion range than the AIS group (11.7 vs. 8.9, *P* < 0.001) and high possibility of fusing to the pelvis (7 vs. 0, *P* = 0.01). Gjolaj et al. recommended extending fusions to the pelvis in patients with Marfan syndrome not only when curves are very distal or dysplastic and associated with pelvic obliquity but also when the distal instrumented vertebrae have suboptimal fixation. In Gjolaj et al.’s report, 3 patients suffered from progression of proximal thoracic curve, 2 of whom underwent revision surgery. Whereas in the AIS group, only 1 patient had a significant progression of an unfused proximal thoracic curve and no revision surgery was needed. Thus, they suggested that the threshold to fuse minor proximal thoracic curves should be lower for patients with Marfan syndrome than for those with AIS [3]. In the current study, no patient in ether group had lower instrumented vertebra (LIV) located at sacrum or pelvis, which was different from that of Gjolaj et al.’s report. And we also analyzed the rates of the patients with L5 instrumented as LIV between the two groups, and no significant difference was found (9.5 %, 4/42 vs. 3.0 %, 5/168, *P* = 0.081). As for the fusion range selection in MSS patients, the criteria were almost the same as that of AIS patients. The average fusion range was 11.5 vertebrae; this was similar to the reports of Lipton and Gjolaj et al. [3, 7] whereas shorter than the report of Di Silvestre and colleagues (average, 12.3, range, 9–17) [9]. However, the average fusion range of our AIS patients was 11.0 vertebrae, which was longer than that of Gjolaj et al.’s report, and this might be the reason why there was no significant difference of the fusion range between the MSS and AIS patients in our study.

In literature, the correction rate in MSS patients was from 36.4 to 60 % [7–12]. In Jones et al.’s 26 cases, the coronal Cobb angle was corrected from 64.5 to 31.6° at final follow-up with an average correction rate of 49.0 % (range, 10.6 ~ 100 %). In their report, the patients with higher correction rate had a high risk of coronal and sagittal decompensation. Thus, they suggested that the correction rate of MSS patients should not be higher than 50 ~ 60 % [8]. Di Silvestre et al. further analyzed subgroup with different fixation system and found that the patients corrected with Harrington and sublaminar wire had an average correction rate of 36.38 % of the coronal Cobb angle (from 70.68 to 45.72°), and the patients corrected with segmental fixation system had an average correction rate of 40.97 %(from 68 to 41.9°), with no significant difference between the two groups [9]. In Gjolaj et al.’s report, the correction rates were 50 and 58 % for the main thoracic curve, and 59 and 60 % for the thoracolumbar/lumbar curve in MSS and AIS patients, respectively, with no significant difference (both *P* > 0.05) [3]. In the current study, the correction rate was higher than 70 %, which might be related to the selection of segmental fixation system, while in the previous reports, Harrington fixation system was also included. In the current study, there was no significant difference of the correction rate of the major curve and this was similar to the report of Gjolaj et al.

Sponseller et al. reported that nearly 40 % Marfan syndrome patients had more than 50° kyphosis [2]. Gjolaj et al. reported that MSS patients had a better thoracolumbar kyphosis correction (−9.5 vs. −0.1°, *P* = 0.05) and better sagittal balance correction (2.4 vs. −0.6 cm, *P* = 0.035) than AIS patients. They thought the reasons might be that the MSS patients had a high incidence of kyphosis, a better flexibility, and a longer fusion range than the AIS patients [3]. In the current study, 3 MSS patients had thoracolumbar kyphosis and all were corrected very well after posterior correction surgery. And the thoracic kyphosis and lumbar lordosis were all maintained very well during follow-up.

Dural ectasia is one of the major diagnostic criteria of Marfan syndrome, which is present in 63–92 % of people with the disorder [10, 13–15]. It can cause thinning of the cortex of the pedicles and the laminae in the lumbosacral spine, thus increased the rate of cerebrospinal fluid leak intraoperatively [3, 8, 16, 17]. However, the prognosis of this complication usually was good with conservative treatment. Some authors reported a higher post-operative wound infection rate in MSS patients, [3, 8] while some others reported different results [7, 9]. Gjolaj et al. also reported Marfan syndrome patients had significantly more instrumentation complications and more reoperations [3]. And Zenner et al. also reported a high complication rate of 30 % in MSS patients [10]. However, in the current study, no patient in either group suffered from cerebrospinal fluid leak or wound infection, and there was no significant difference according to the complication rate between the two groups. This may be in part due to the fact that the patients in the current study underwent only posterior correction surgery while in the other reports, both anterior surgery and posterior surgery were included.

## Conclusions

From this retrospective study with small sample of Marfan syndrome scoliosis patients, we found that when posterior scoliosis correction surgery was selected, the Marfan syndrome scoliosis patients had similar correction results, while having longer operation duration and more blood loss compared with adolescent idiopathic scoliosis.

## References

[1] Pyeritz RE, Francke U (1993). The second international symposium on the Marfan syndrome. Am J Med Genet.

[2] Sponseller PD, Bhimani M, Solacoff D, Dormans JP (2000). Results of brace treatment of scoliosis in Marfan syndrome. Spine.

[3] Gjolaj JP, Sponseller PD, Shah SA, Newton PO, Flynn JM, Neubauer PR (2012). Spinal deformity correction in Marfan syndrome versus adolescent idiopathic scoliosis: learning from the differences. Spine.

[4] De Paepe A, Devereux RB, Dietz HC, Hennekam RC, Pyeritz RE (1996). Revised diagnostic criteria for the Marfan syndrome. Am J Med Genet.

[5] The Working Group on 3-D Classification (Chair Larry Lenke, MD), and the Terminology Committee. SRS Terminology Committee and Working Group on Spinal Classification: revised glossary of terms. Available at: http://www.srs.org/professional/glossary/SRS_revised_glossary_of_terms.htm Accessed Nov 29, 2011.

[6] Thompson JP, Transfeldt EE, Bradford DS, Ogilvie JW, Boachie-Adjei O (1990). Decompensation after Cotrel-Dubousset instrumentation of idiopathic scoliosis. Spine.

[7] Lipton GE, Guille JT, Kumar SJ (2002). Surgical treatment of scoliosis in Marfan syndrome: guidelines for a successful outcome. J Pediatr Orthop.

[8] Jones KB, Erkula G, Sponseller PD, Dormans JP (2002). Spine deformity correction in Marfan syndrome. Spine.

[9] Di Silvestre M, Greggi T, Giacomini S, Cioni A, Bakaloudis G, Lolli F (2005). Surgical treatment for scoliosis in Marfan syndrome. Spine.

[10] Zenner J, Hitzl W, Meier O, Auffarth A, Koller H (2014). Surgical outcomes of scoliosis surgery in Marfan syndrome. J Spinal Disord Tech.

[11] Li ZC, Liu ZD, Dai LY (2011). Surgical treatment of scoliosis associated with Marfan syndrome by using posterior-only instrumentation. J Pediatr Orthop B.

[12] Li QY, Qiu GX, Wang YP, Zhang JG, Shen JX, Weng XS (2005). Clinical presentation and surgical treatment of scoliosis in Marfan syndrome. Chin Med J (Engl).

[13] Pyeritz RE, Fishman EK, Bernhardt BA, Siegelman SS (1988). Dural ectasia is a common feature of the Marfan syndrome. Am J Hum Genet.

[14] Villeirs GM, Van Tongerloo AJ, Verstraete KL, Kunnen MF, De Paepe AM (1999). Widening of the spinal canal and dural ectasia in Marfan’s syndrome: assessment by CT. Neuroradiology.

[15] Fattori R, Nienaber CA, Descovich B, Ambrosetto P, Reggiani LB, Pepe G (1999). Importance of dural ectasia in phenotypic assessment of Marfan’s syndrome. Lancet.

[16] Stern WE (1988). Dural ectasia and the Marfan syndrome. J Neurosurg.

[17] Sponseller PD, Ahn NU, Ahn UM, Nallamshetty L, Rose PS, Kuszyk BS (2000). Osseous anatomy of the lumbosacral spine in Marfan syndrome. Spine.

